# Automatic image annotation method based on a convolutional neural network with threshold optimization

**DOI:** 10.1371/journal.pone.0238956

**Published:** 2020-09-23

**Authors:** Jianfang Cao, Aidi Zhao, Zibang Zhang

**Affiliations:** 1 School of Computer Science & Technology, Taiyuan University of Science and Technology, Taiyuan, China; 2 Department of Computer Science & Technology, Xinzhou Teachers University, Xinzhou, China; Politechnika Slaska, POLAND

## Abstract

In this study, a convolutional neural network with threshold optimization (CNN-THOP) is proposed to solve the issue of overlabeling or downlabeling arising during the multilabel image annotation process in the use of a ranking function for label annotation along with prediction probability. This model fuses the threshold optimization algorithm to the CNN structure. First, an optimal model trained by the CNN is used to predict the test set images, and batch normalization (BN) is added to the CNN structure to effectively accelerate the convergence speed and obtain a group of prediction probabilities. Second, threshold optimization is performed on the obtained prediction probability to derive an optimal threshold for each class of labels to form a group of optimal thresholds. When the prediction probability for this class of labels is greater than or equal to the corresponding optimal threshold, this class of labels is used as the annotation result for the image. During the annotation process, the multilabel annotation for the image to be annotated is realized by loading the optimal model and the optimal threshold. Verification experiments are performed on the MIML, COREL5K, and MSRC datasets. Compared with the MBRM, the CNN-THOP increases the average precision on MIML, COREL5K, and MSRC by 27%, 28% and 33%, respectively. Compared with the E2E-DCNN, the CNN-THOP increases the average recall rate by 3% on both COREL5K and MSRC. The most precise annotation effect for CNN-THOP is observed on the MIML dataset, with a complete matching degree reaching 64.8%.

## Introduction

With the continued development of network technology and the growing popularity of multimedia devices, network image data are growing at an exponential rate. Taking WeChat (a communication software) as an example, the daily number of uploaded images in WeChat moments now exceeds a hundred million [1]. In this information explosion era, organizing and retrieving unlabeled images has become a research interest in the field of image management [2]. Unlike previous single-label classified images, most images currently contain rich semantic content, where a common image normally contains several keywords or labels [3]. On the one hand, artificial annotation has low efficiency, and it can only complete annotation for a limited number of images. On the other hand, although artificial annotation achieves relatively high annotation accuracy, its outcomes are likely to be affected by subjective elements. Furthermore, the manpower and time costs are high. To solve these problems, experts and scholars have proposed automatic multilabel image annotation, namely, assigning multiple labels to an image that contains rich semantic information to complete image annotation by computer [4, 5]. These labels, covering multiple image semantics, can be used to reasonably and effectively manage these image data. Thus, the massive number of images in these networks can be better used and analyzed, and the subjective errors and costs of artificial annotation can be greatly reduced.

Based on the aforementioned information, this study focuses on automatic multilabel image annotation based on deep learning.

## Related works

Automatic image annotation can be widely applied in the fields of image retrieval and image classification. To date, a number of automatic annotation methods have been proposed, and these methods can be roughly divided into two categories: one is based on traditional machine learning, and the other is based on deep learning. For the traditional machine learning-based category, Zou et al. [6] proposed a multiview multilabel (MVML) learning algorithm, integrating multifeature (view) and ensemble learning simultaneously and utilizing the complementarity among the views and the base learners of ensemble learning to improve the annotation accuracy. The accuracy can be enhanced by integrating multiple classifiers used for prediction. However, the training process of the model may be complex and inefficient. Tan et al. [7] proposed an approach called multilabel classification based on low-rank representation (MLC-LRR). First, a low-rank constrained coefficient matrix is calculated by using low-rank representation in the image feature spaces. Then, a feature-based graph is defined, the global relationship between images is captured, and a semantic graph is constructed. Finally, the multilabel classifier is trained by combining these two graphs. However, this method hinges heavily on the proportion of annotated images and does not consider the semantic differences between the underlying features and high-level features. Hu et al. [8] proposed a MIML-KNN method based on metric learning. The Laplacian matrix label is learned to derive the label correlations by minimizing the label manifold regularizer. They proposed a novel MIML objective function and constructed the MIML-KNN classifier using Hausdorff distances. This method calls for large amounts of calculation for massive training data. If there are wrong data beside values of the classified class, classification errors and poor fault tolerance will be introduced to the training data. Yang et al. [9] proposed automatic image annotation based on multiview deep representation. To process various keywords and select appropriate features in the image, they suggested a multiview stacked autoencoder (MVSAE) framework, which was used to establish a correlation between the underlying visual features and the high-level semantic information to realize automatic image annotation. Tian et al. [10] discussed a Gaussian mixture model (GMM)-based automatic image annotation method. They used the GMM for training model construction and rival penalized expectation maximization (RPEM) for posterior probability assessments. Additionally, they constructed label similarity graphs to avoid polysemy occurring during annotation and then used the rank-two relaxation heuristics algorithm to deeply explore the correlation between candidate labels. Joshua et al. [11] proposed an automatic image annotation method based on a multiclass support vector machine with hybrid kernels. They used the linear binary pattern-discrete wavelet transform (LBP-DWT) technique to extract image features from the horizontal, vertical and diagonal directions. Tang et al. [12] proposed a semisupervised adaptive hypergraph learning method for automatic image annotation, and they used limited annotated data and abundant unlabeled data to improve annotation performance. However, due to the existence of semantic gaps, the annotation effect is still undesirable for images with complex backgrounds. The above traditional machine learning methods usually extract features by manual labor during the image feature extraction process, which unavoidably gives rise to subjective errors, resulting in errors in image information extraction and poor experimental accuracy.

In recent years, deep learning-based methods have become a major focus in research on automatic image annotation [13]. Image features are extracted by convolution operations [14], and the relationship between image features and labels is set up by using a deep neural network training model. In 2006, Hinton [15] first proposed to effectively train features in a training set using a deep neural network. Later, Markatopoulou et al. [16] proposed a deep convolutional neural network (DCNN) architecture in which the trained DCNN was treated as an independent classifier to evaluate the direct output of the whole network and train it as a feature generator. Wang et al. [17] developed a dual model based on the multilabel selection algorithm, integrating a discriminative model with a nearest-neighbor-based model. Laib et al. [18] proposed a potential topic model based on a latent Dirichlet allocation and a convolutional neural network for event classification and image annotation. Based on initial labels extracted from the CNNs and initial labels of possibly user-defined tags, the event categories and final annotations of the images were estimated through a refinement process based on the expectation-maximization (EM) algorithm. Ke et al. [19] developed an end-to-end automatic image annotation model based on a deep convolutional neural network (E2E-DCNN) and multilabel data augmentation. A deep CNN structure was adopted for adaptive feature learning, in which the cross-entropy loss functions were first used to construct an end-to-end annotation structure for training, and Wasserstein generative adversarial networks were used for multilabel data augmentation. The deep neural network model has made headway in the field of image labeling. However, there are still some deficiencies. The deep learning annotation models represent improvements in the models themselves. However, for the semantic content of different images, differences between images are not fully considered. Annotation should be treated indiscriminately whether the method of setting the threshold or the ranking function is used to label the test images. The same threshold, such as 0.5, is set for each label, or the ranking function is used to uniformly assign the first few probability labels. As a result, multiple or fewer labels occur when the label number of the images is unknown. The deep neural network is a model containing multilayer nonlinear operations. It has a powerful representation ability and can learn many complex structures. However, a more complex structure may easily result in overfitting [20].

To solve the problems of multiple or fewer labels and overfitting caused by deep networks, this study proposes a convolutional neural network with threshold optimization (CNN-THOP). First, to speed up the training speed of CNNs and prevent overfitting to some extent, batch normalization (BN) [21] is added before the activation layer of traditional CNNs. The purpose of introducing BN is to realize standardization and linear transformation for data, which enables the activated input values to fall within a domain that is sensitive to inputs. Thus, a small change can cause an increased gradient, thereby preventing the problem of gradient loss during backpropagation. Next, the CNN is constructed to learn image features, and a backpropagation algorithm is used to train the model to obtain parameters. Finally, the model obtained by training is used to predict the test set to obtain a probability matrix. Then, threshold optimization is performed on the probability matrix. Based on the comparisons of label correlations under different thresholds with the corresponding actual label, an optimal threshold for each class of labels is determined. Compared with previous methods using a fixed threshold and fixed label number, the threshold optimization method in this study provides more flexible and precise image annotation.

The novelty of this study is as follows:

1. A BN layer is added into the CNN model. To solve the problem of the gradient loss of the bottom network during backpropagation due to an increase in the number of network layers, this study introduces a BN layer between the convolution layer and the activation layer to standardize the data before entering the next layer of the network. This treatment enables the feature values of each layer to fall within the domain where the activation function is sensitive. Thus, even a small change can cause the loss function to produce a great change.
2. An effective threshold optimization algorithm is proposed. To avoid the drawbacks of a possible empty label set for some images due to a fixed threshold or overlabeling or downlabeling caused by the Top k algorithm, this study proposes a threshold optimization algorithm. Using this method, each class of labels is separately analyzed. The optimal threshold for each class is determined based on the correlations of the predicted labels under different thresholds with the authentic labels. Then, annotation is completed with a label whose prediction probability is no less than the optimal threshold. This addition addresses the problems faced by the fixed threshold method and the Top k algorithm, realizes flexible annotation and improves annotation accuracy.

## Methods

### Convolutional neural network

A convolutional neural network [22] (CNN), a kind of feedforward neural network, is essentially a multilayer perceptron. It was proposed by Hubel and Wiesel [23] in the 1960s in their research on neurons used for local sensitivity and direction selection in the cerebral cortex of cats, and breakthroughs were then made by Cun et al [24] on the MNIST handwritten digital dataset. The main architecture of CNNs includes an input layer, a convolutional layer, a pooling layer, a fully connected layer and a final output layer. The number of network layers is deepened by superposing the convolutional layer and pooling layer. The local connection and weight sharing method adopted by CNNs discards some neurons, thereby reducing the risk of overfitting. However, parameter sharing among different neurons decreases the number of weights, making the network easier to optimize. A CNN automatically extracts image features through the convolutional operation of the convolutional layer, which reduces the error rate of information loss compared with artificial feature extraction in the traditional method and has achieved great success in the vision field of computers. The convolutional layer, the core of a CNN, extracts features from the output of the previous layer in the CNN. During this process, multiple convolution kernels are used for convolutional operations to finally obtain multiple feature maps. The convolutional operation formula is shown in Formula (1) [25]:

$$y_{l}^{i}=f\left(\sum_{l\in M_{j}}y_{l}^{i-1}*k_{j}^{i}+b_{j}^{i}\right),$$ (1)

where $y_{j}^{i}$ represents the feature map output by the *j*th convolutional kernel at the *i*th layer; *M*_*j*_ represents all the feature maps at the *i*−1 layer; $k_{j}^{i}$ represents a convolutional kernel at the *i*th layer; $b_{j}^{i}$ represents the bias corresponding to the $y_{j}^{i}$ features at the *i*th layer; *f*() represents the function operation; and * represents the convolution operation. To obtain as many features as possible, multiple convolutional kernels are used during the convolution process, which inevitably causes information redundancy. To reduce the feature dimension, a pooling operation is adopted after convolution. At present, the commonly used pooling operations include max-pooling and mean-pooling. After the pooling process, the dimensions of the feature maps are reduced. As max-pooling can retain the texture information of the image well, the max-pooling method is adopted in this study.

The loss function measures the difference degree between the predicted value and the real value for the output. For a binary classification problem, the sigmoid activation function and cross-entropy loss function are usually adopted at the output layer to calculate the value of the prediction label. The multilabel annotation problem in this study can be transformed into a binary classification problem on each label. Therefore, the output layer also adopts the sigmoid activation function to calculate the predicted value, and each label is independently distributed and free from mutual influence. Therefore, the binary cross-entropy loss function is used as the loss function of the network model in this study, as in Formula (2) [26]:

$$\mathrm{loss}=-\sum_{i=1}^{n}\left[y_{i}\log{\hat{y}}_{i}+(1-y_{i})\log(1-{\hat{y}}_{i})\right],$$ (2)

where *n* is the number of labels, *y*_*i*_ is the real value of the *i*th class labels, ${\hat{y}}_{i}$ is the predicted value of the *i*th class labels, and *loss* is the loss function of a single sample.

### CNN model fusing the threshold optimization

**Fig 1:**
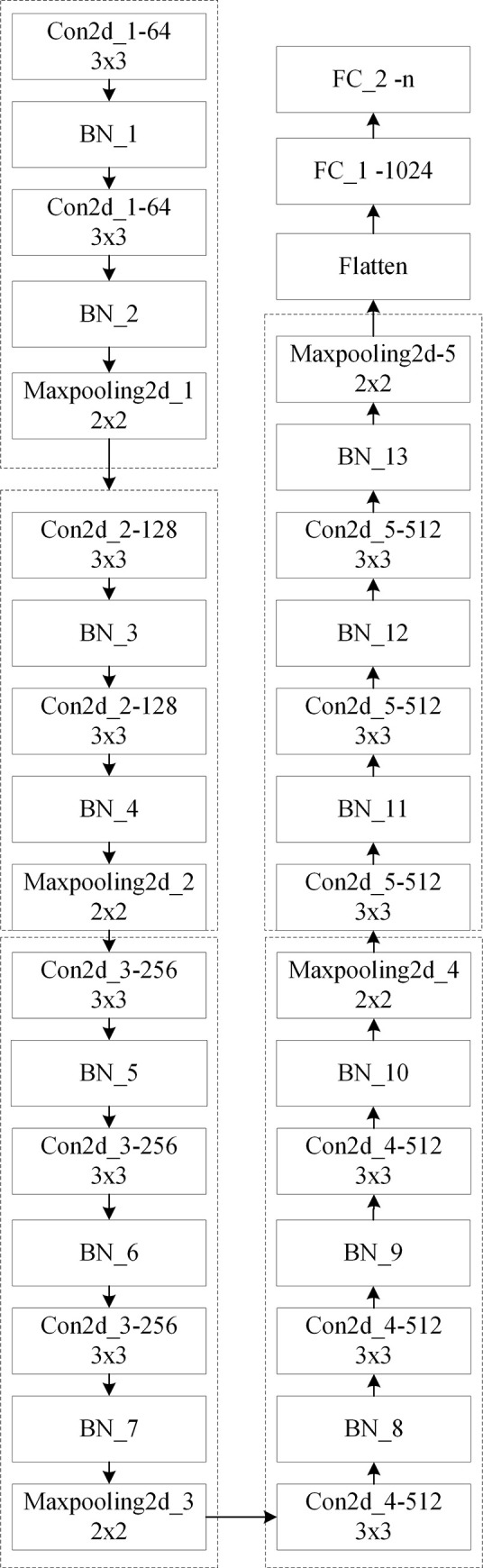
CNN architecture used in this study.

A number of mature CNN networks have been used for feature extraction, including AlexNet [24], VGGNet [27], GoogLeNet [28] and ResNet [29], which have been considered to reach a new height of effectiveness in image classification. Considering that VGGNet has a simpler architecture than GoogLeNet and ResNet but with sufficient width and depth for feature extraction, we use VGG16 as the base for model improvement. The architecture of the CNN adopted in this study is shown in Fig 1.

The input layer of the network model consists of images of the same size, all of which are 224×224×3, where 3 represents three channels, i.e., R, G and B. The middle processing layer includes five groups of convolutions, in an imitation of the VGG16 network architecture. After each group of convolutions, a max-pooling is connected. A total of five pooling layers are adopted, and BN is added before each activation function to speed up the convergence. The convolutional kernels used in the five groups of convolutional operations are the classic 3×3 size, and the number of convolutional kernels is 64, 128, 256, 512, and 512. The pooling layer adopts max-pooling, and the pooling windows are all a 2×2 size. A dropout operation is conducted to prevent overfitting, and the probability is set to 0.5. Subsequently, a flattening operation is performed to flatten the data for full connection. In the final output layer, there are two fully connected layers. The first one uses 1024 nodes, and the number of nodes adopted in the last fully connected layer is flexibly designed according to the number of classes designed in the dataset. In the whole network structure, only the activation function of the last output layer adopts the sigmoid activation function, and the other activation functions all adopt a rectified linear unit (ReLU). The optimizer adopts a stochastic gradient descent (SGD). The initial value of the learning rate is 0.005, and the learning rate automatically updates and decreases.

### Modifications

#### Accelerated convergence speed based on BN

The unsteady input distribution of the hidden layer neuron during the training process of the deep neural network prevents the network from learning in a stable way. Meanwhile, with the deepening of the network layer, the distribution of the activated input value gradually deviates, approaching the limited saturated zone of the nonlinear function and causing the gradient to minimize until it disappears during backpropagation. When training a new batch of data, the network is required to relearn the characteristics of this batch, meaning that with the deepening of the network layer number, the gradient gets smaller and the training becomes increasingly difficult, with a slower convergence speed. Therefore, this study integrates the BN [21] to fix the activation distribution of each hidden layer neuron and standardize the output of the neurons on the previous layer (normal distribution). With this step, the activation input value can be pulled back to the linear area from the nonlinear area, thereby increasing the derivative value and enhancing the gradient. Meanwhile, to improve the expression ability of the network, another transformation is carried out and then input to the neuron at the next layer. In this way, we can address the training data increasingly deviating with the deepening of layers and fix the distribution of input data for each layer of the network at the same time so that the activated input values all fall within an area sensitive to the input. Therefore, a small change will be responsible for a major change in the loss function in that the increased gradient can greatly accelerate the convergence speed, thereby addressing the gradient of the underlying network disappearing during backpropagation.

The significance of BN is to standardize the data output from the preceding layer of the network. During the standardization process, the average of each small batch of activation values that are input into the network has to be calculated. The calculation equation is as follows [21]:

$$\mu_{B}=\frac{1}{m}\sum_{i=1}^{m}x_{i};$$ (3)

Based on the small batch average, the variance of the activation values for the small batch is calculated as follows [21]:

$$\sigma_{B}^{2}=\frac{1}{m}\sum_{i=1}^{m}(x_{i}-\mu_{B}{)}^{2};$$ (4)

Based on the obtained average and variance, the data for the small batch are standardized, and the standardization equation is as follows [21]:

$$\hat{x_{i}}=\frac{x_{i}-\mu_{B}}{\sqrt{\sigma_{B}^{2}+\varepsilon }};$$ (5)

To improve the representation ability of the network, the standardization outcomes are subjected to linear transformation, and the equation is as follows [21]:

$$y_{i}=\gamma \hat{x_{i}}+\beta \equiv BN_{\gamma ,}{}_{\beta }(x_{i}),$$ (6)

where *x* is the activation value of a hidden layer neuron before transformation, *m* is the number of instances in the batch processing, *μ*_*B*_ is the mean value of *m* instances in the batch training, $\sigma_{B}^{2}$ is the variance of *m* instances in the batch training, $\hat{x_{i}}$ is the transformation created by subtracting the mean of *m* instances in the batch processing from the original activation *x* corresponding to a neuron before the result is divided by the variance, *ε* is the error, *γ* and *β* are parameters learned during the training stage, and *y*_*i*_ is the normalized network response.

The specific BN operations are as follows:

Input: Sample *x*: *B* = {*x*_1…*m*_} to be entered into the activation function, and parameters to be learned: *γ* and *β*

Output: Normalized network response *y*_*i*_;

Step 1: Calculate the sample mean value *μ*_*B*_; Step 2: Calculate the sample variance $\sigma_{B}^{2}$;
Step 3: Standardize the sample data to obtain $\hat{x_{i}}$;
Step 4: Continuously iterate and train parameters *γ* and *β*, output *y*, and obtain the new value, i.e., *y*_*i*_, through the linear transformation of *γ* and *β*.

In the network test stage, the activation *x* of a neuron can form a normal distribution, which approximates the linear area of the nonlinear area, thereby augmenting the derivative value and the backpropagation mobility and accelerating the network convergence. Furthermore, to improve the representation ability of the network, another linear transformation is performed, and the BN for the test samples is as follows:

$$y=\frac{\gamma }{\sqrt{Var[x]+\varepsilon }}x+(\beta -\frac{\gamma E[x]}{\sqrt{Var[x]+\varepsilon }}),$$ (7)

where *γ* and *β* are parameters learned during the training stage, *E*[*x*] is the mean for all batches (the number of images set in the training network) during the training stage, and *Var*[*x*] is the unbiased estimation for the variance of each variance.

### Optimal threshold set by fusing threshold optimization

Previously, a fixed threshold (e.g., 0.5) or Top k (e.g., k = 5) was normally used to determine the label assignment for automatic image annotation. A serious drawback of using a fixed threshold is that it can result in an empty label set for some images when the prediction probabilities of the labels are all lower than the preset threshold. On the other hand, Top k considers the differences of images in content and semantics; that is, it assigns an equivalent number of labels to all images, which may lead to overlabeling or downlabeling. Targeting these fixed threshold and Top k problems, a threshold optimization algorithm is used in this study to set an optimal threshold for each class of labels.

The CNN is used to test images in the test set and obtain an array of probabilities. The array element is the prediction probability for each class of label for each image. It is necessary to set an optimal threshold for each class of label to determine whether this label is assigned to the said image. The threshold optimization algorithm designed in this study is described as follows:

Input: The test set’s label probability array predicted by the model
Output: The *best_ threshold[]*
Step 1: Initialization of parameters: Define the variables *index* and array *a*, define the array *threshold* and set its range [0.1,0.8], where the step is 0.1, and then initialize the *best_ threshold*[0 0 0 0 0];
Step 2: Read the *i*-th column element (*i* starts from 1) *out*, which represents the *i*-th label of all images in the test set and is labeled *y_ prob*;
Step 3: Take the *j*-th value in the *threshold* (*j* starts from 1) and compare with elements in *y_prob* in turn. If it is greater than or equal to *j*, it is set to 1; otherwise, it is 0 and recorded as *y_ pred*;
Step 4: Perform the Matthews operation on the forecast label *y_pred* and real label *y_test[*:,*i]* to obtain the operation result *a*;
Step 5: Repeat steps 2 and 3 until *j* is traversed;
Step 6: Calculate the *index* for the position of the maximum in *a*;
Step 7: Take out the threshold *best_threshold[i]* corresponding to the *index* position in the *threshold* as the threshold of the *i*th class label;
Step 8: Repeat Steps 2–7 until all labels are traversed.

It is worth mentioning that the Matthews correlation coefficient function is used in step 4. Compared with other correlation coefficients, such as Pearson’s correlation coefficient, the Matthews correlation coefficient considers the real and false positivity and negativity of the label. In addition, this coefficient is not affected by the imbalance of datasets and therefore serves as a balanced criterion of measurement. It is one of the best correlation assessment methods in the field of image classification.

**Fig 2:**
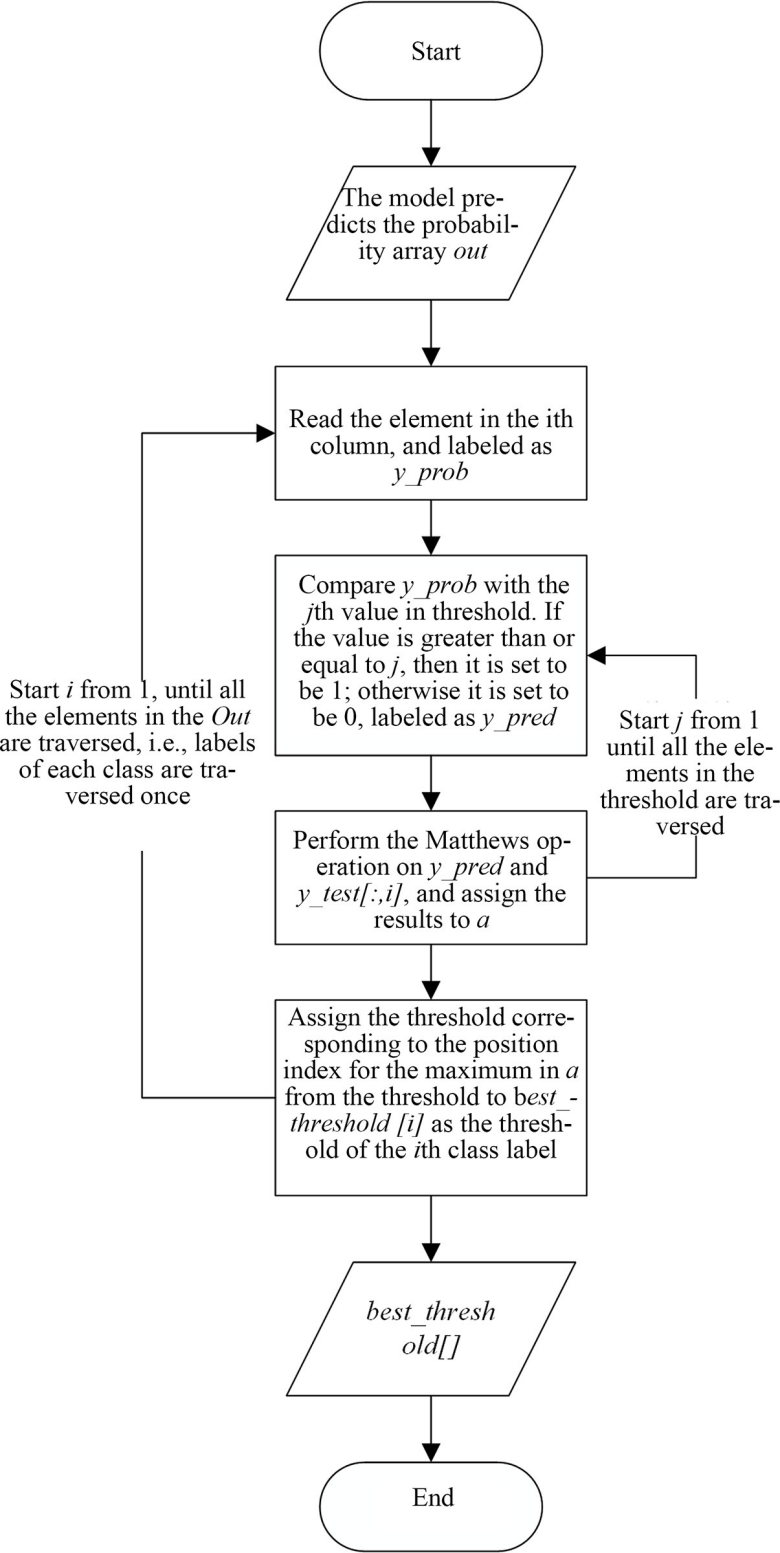
Flow chart of threshold optimization.

The basic flow chart of threshold optimization is shown in Fig 2.

### Multilabel image annotation framework

**Fig 3:**
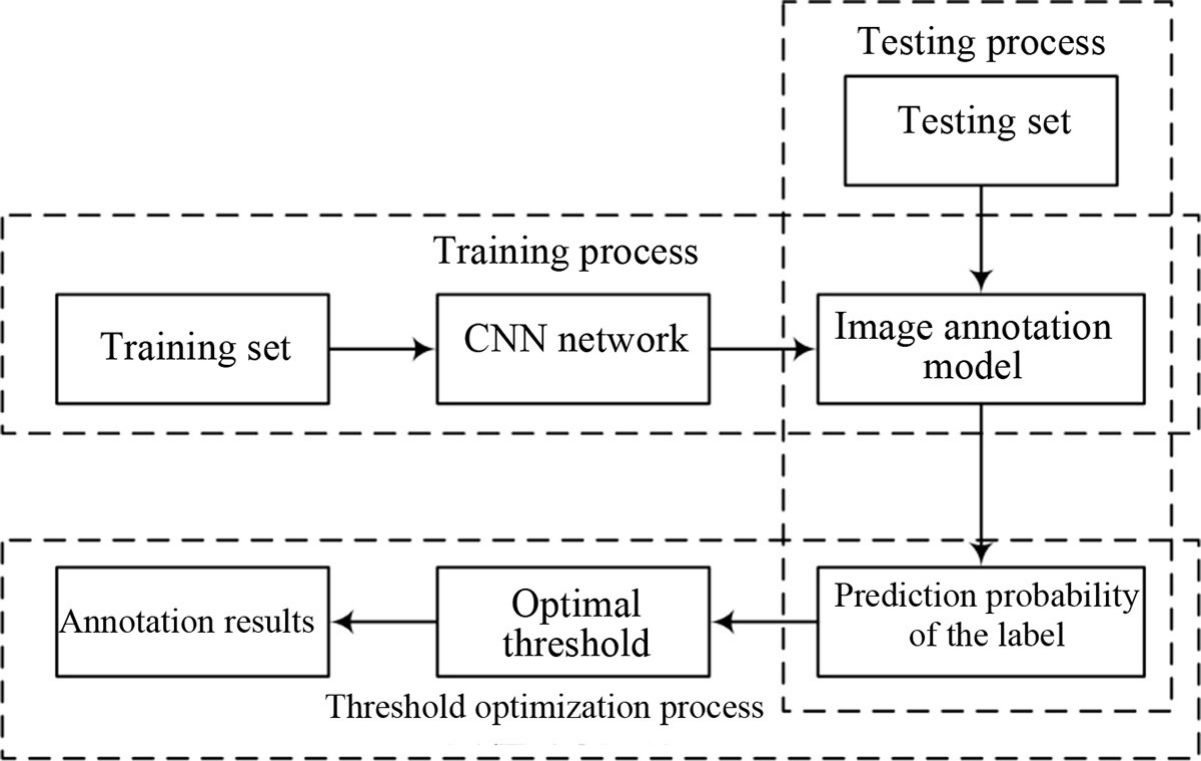
The multilabel automatic image annotation framework.

The multilabel automatic image annotation framework designed in this study is shown in Fig 3.

First, the training dataset is input into the CNN for training to obtain a labeling model. In this study, BN is added before the activation layer of the CNN to accelerate the convergence speed. Second, the trained model is used to predict the test dataset to obtain the prediction probability for the label. Then, the threshold optimization algorithm is employed for the threshold optimization of the prediction probability to obtain the best threshold, which, to a certain extent, solves the problem of overlabeling or downlabeling caused by a fixed number of labels. Finally, labels corresponding to the optimal threshold are used to label images to obtain the final annotation results.

## Experiment and discussion

### Experimental data

To verify the effectiveness of the CNN-THOP proposed in this study for image annotation, we use free, publicly available datasets: MIML [30] on natural scenes provided by Learning and Mining from Data (LAMDA) of Nanjing University, COREL5K [31] collated by the Corel Company and MSRC [32] from Microsoft Research Cambridge. Details of the datasets are shown in Table 1.

**Table 1:** Related information of three datasets.

Dataset	#Images	#Classes	#Training images	#Test images	#Average label number per image
MIML	2000	5	1600	400	1.2
COREL5K	5000	260	4500	500	3.5
MSRC	591	22	472	119	2.5

### Experimental design

In this study, we conduct an image annotation simulation experiment based on the deep learning library Keras. To objectively evaluate the experimental results, we use a variety of evaluation indexes, including the average precision *AP*, average recall rate *AR* and *F1*. Meanwhile, to explain the annotation effect more accurately, a new evaluation index, the complete matching degree (*CMD*), is defined in this study as the degree to which the result of the labeled word tested is completely consistent with the real labeled word of the image when testing each picture as another evaluation standard of the experimental result.

## Results analysis

### Experimental verification of BN operation

**Fig 4:**
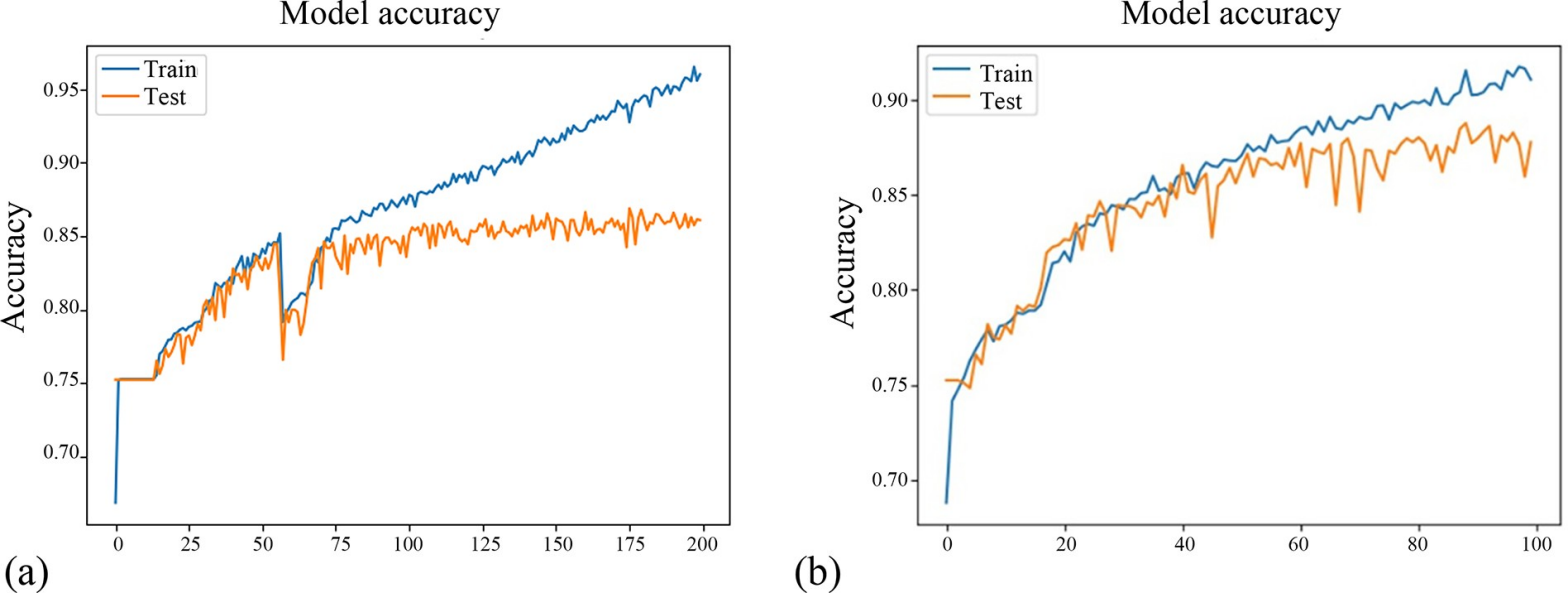
Comparison of variation in iteration accuracy. (a) Model without BN. (b) Model with BN.

To verify that an added BN can accelerate the convergence speed, we use the MIML dataset to train the network architecture with an added BN and the architecture without an added BN. With the increase in the number of iterations, the variation in accuracy is shown in Fig 4.

Due to the differences in training duration, we conduct 200 iterations for the network with an added BN and 100 iterations for the network structure without a BN to illustrate the role of BN. The abscissa in Fig 4(A) and 4(B) is Epoch, and the ordinate is Accuracy. As shown in Fig 4(A), the accuracy rate for approximately 40 iterations reaches 80% without an added BN, while the accuracy rate for 20 iterations only reaches 75%. As shown in Fig 4(B), with an added BN, it only requires approximately 15 iterations for the accuracy to reach 80% and 5 iterations to reach 75%, indicating that BN can greatly accelerate the convergence speed.

### Verification of the optimal threshold

Given the relatively large number of label classes in the COREL5K and MSRC datasets and the numerous thresholds involved, it is inconvenient to display them one by one, so an ellipsis is used here. For the MIML dataset, as only 5 classes of labels are involved, all of them are selected for display. The above three datasets are used to verify the effectiveness of the CNN-THOP proposed in this study in setting the optimal threshold for each class of labels. To find the optimal threshold, experiments are carried out to compare each group of thresholds. Each group of thresholds in Table 2 are the optimal thresholds corresponding to the models with the same iterations. The optimal threshold is detected by loading the optimal threshold corresponding to each model into the optimal model.

**Table 2:** Experimental results for each group of thresholds.

Dataset	threshold	*AP*	*AR*	*AF1*	*CMD*
MIML	[0.6 0.1 0.3 0.1 0.6]	0.800	0.776	0.783	0.625
	[0.8 0.7 0.5 0.2 0.3]	0.824	0.739	0.775	0.630
	[0.7 0.1 0.6 0.2 0.8]	0.819	0.754	0.781	0.632
	[0.6 0.5 0.1 0.1 0.7]	0.816	0.771	0.786	0.645
	[0.3 0.3 0.2 0.1 0.7]*	0.807	0.776	0.787	0.648
COREL5K	[0.2 … 0.4 … 0.3]	0.536	0.507	0.521	0.402
	[0.4 … 0.1 … 0.6]	0.524	0.473	0.497	0.386
	[0.8 … 0.2 … 0.5]	0.515	0.481	0.497	0.371
	[0.6 … 0.2 … 0.7]	0.501	0.456	0.477	0.359
	[0.1 … 0.3 … 0.5]*	0.527	0.583	0.553	0.412
MSRC	[0.2 … 0.4 … 0.6]	0.742	0.689	0.714	0.561
	[0.5 … 0.3 … 0.7]	0.723	0.662	0.691	0.534
	[0.8 … 0.6 … 0.1]	0.707	0.716	0.756	0.493
	[0.3 … 0.4 … 0.2]	0.760	0.724	0.741	0.578
	[0.1 … 0.3 … 0.5]*	0.761	0.783	0.771	0.586

* indicates the optimal threshold for this class of dataset

As shown in Table 2, the newly added evaluation index *CMD* makes the evaluation of experimental results more firm and rigorous. First, the overall analysis of the experimental results for each group of thresholds reveals that the difference is not large, indicating that the optimal model obtained in the training has a good effect. Therefore, it is generally believed that this CNN model is not problematic for the automatic annotation of multilabel images in this dataset. Second, although the difference between the results obtained for each group of thresholds is not large, considering that the *CMD* is the highest, thresholds marked with an asterisk in Table 2 are selected as the best threshold for each dataset. Among them, *CMD* reaches 64.8%, 41.2% and 58.6% in the three datasets in this experiment, indicating the effectiveness of the optimal threshold once again.

### Comparison with other CNNs

In this study, we use a modified VGG16 as the image feature extractor. We investigate the influence of different CNN architectures on the experimental outcomes based on three datasets. The results are summarized in Table 3.

**Table 3:** Comparison among different CNN architectures.

Dataset	CNN architecture	*AP*	*AR*	*F1*
MIML	AlexNet	0.683	0.675	0.678
	VGG16	0.756	0.731	0.743
	ResNet101	0.769	0.774	0.771
COREL 5K	AlexNet	0.310	0.384	0.343
	VGG16	0.364	0.432	0.395
	ResNet101	0.372	0.459	0.410
MSRC	AlexNet	0.593	0.614	0.603
	VGG16	0.687	0.695	0.691
	ResNet101	0.432	0.407	0.419

As shown in Table 3, on datasets with an appropriate size, the experimental results improve as the network architecture deepens. This is because deeper-level architecture can extract higher-level features. On MSRC, however, a deeper-level architecture (ResNet101) does not perform better. This is possibly because the scale of the dataset is limited, whereas the network layers are too deep, which leads to overfitting. Therefore, we use VGG16 for modification to construct the network architecture.

### Comparison with other image annotation methods

Since it is inconvenient to fully display the hundreds of classes involved in the three datasets, we randomly extract some classes from these three datasets and conduct experiments to compare the accuracies among the CNN-THOP proposed in this study and the traditional multiple Bernoulli relevance model [33] (MBRM) and spatial spectrum kernel [34] (SSK) combined with the context-based keyword propagation [35] (CBKP) (SSK+CBKP), and the deep learning method commonly used in recent years based on the convolutional neural network and adaptive thresholding [36] (CNN-AT), a multilabel semantic image annotation approach combining a deep convolutional neural network and ensemble of classifier chains [37] (CNN-ECC), the end-to-end automatic image annotation model based on a deep convolutional neural network [19] (E2E-DCNN) and a method in the literature [38]. The results are shown in Table 4.

**Table 4:** Comparison of annotation precision for each class in different datasets using different algorithms.

Dataset	Label class	Annotation precision						
		MBRM	SSK+CBKP	CNN-AT	CNN-ECC	E2E-DCNN	Literature [38]	CNN-THOP
MIML	Desert	0.813	0.862	0.915	0.908	0.953	0.965	0.982
	Mountain	0.769	0.804	0.806	0.825	0.814	0.824	0.866
	Sea	0.713	0.796	0.798	0.804	0.835	0.896	0.890
	Tree	0.735	0.813	0.809	0.812	0.876	0.923	0.939
	Sunset	0.862	0.789	0.781	0.797	0.850	0.980	0.991
COREL5K	jet	0.700	0.765	0.806	0.794	0.801	0.821	0.825
	fox	0.753	0.815	0.841	0.849	0.864	0.892	0.859
	plane	0.736	0.794	0.835	0.856	0.891	0.885	0.904
	snow	0.749	0.806	0.836	0.828	0.896	0.904	0.937
	sky	0.715	0.783	0.817	0.815	0.842	0.854	0.875
	arctic	0.652	0.734	0.772	0.773	0.785	0.773	0.762
	Sunset	0.762	0.801	0.834	0.825	0.858	0.853	0.873
	train	0.815	0.916	0.950	0.963	0.967	0.982	0.971
	flower	0.864	0.925	0.949	0.950	0.963	0.956	0.982
	bus	0.634	0.714	0.734	0.745	0.752	0.747	0.735
	beach	0.612	0.688	0.690	0.694	0.683	0.692	0.648
	dinosaur	0.553	0.602	0.598	0.562	0.625	0.635	0.617
	nest	0.651	0.709	0.732	0.752	0.751	0.752	0.764
	bird	0.857	0.918	0.948	0.957	0.964	0.965	0.987
	wood	0.796	0.860	0.881	0.896	0.906	0.927	0.935
	dog	0.805	0.859	0.894	0.904	0.928	0.958	0.962
	cliff	0.602	0.668	0.682	0.714	0.701	0.712	0.687
	people	0.876	0.924	0.956	0.977	0.975	0.979	0.980
	track	0.809	0.853	0.870	0.875	0.861	0.862	0.891
	car	0.869	0.928	0.952	0.953	0.954	0.942	0.972
	water	0.815	0.886	0.904	0.922	0.928	0.935	0.956
	tree	0.839	0.864	0.916	0.933	0.943	0.942	0.972
	polar	0.750	0.783	0.799	0.846	0.855	0.861	0.864
	grass	0.726	0.765	0.799	0.807	0.829	0.832	0.866
	bear	0.731	0.788	0.807	0.815	0.838	0.844	0.880
MSRC	grass	0.734	0.762	0.816	0.852	0.865	0.902	0.931
	cow	0.755	0.812	0.880	0.889	0.894	0.899	0.916
	tree	0.736	0.786	0.841	0.850	0.865	0.951	1
	sky	0.724	0.804	0.864	0.866	0.887	0.852	0.848
	building	0.734	0.795	0.853	0.862	0.890	0.958	0.96
	aeroplane	0.703	0.765	0.829	0.837	0.846	0.895	0.923
	mountain	0.665	0.728	0.765	0.798	0.804	0.822	0.75
	face	0.847	0.908	0.955	0.951	0.965	0.900	0.888
	body	0.716	0.764	0.908	0.914	0.936	0.896	0.857
	car	0.809	0.855	0.900	0.911	0.938	0.952	1
	bike	0.759	0.803	0.856	0.863	0.890	0.942	1
	sheep	0.718	0.768	0.815	0.827	0.865	0.873	0.889
	flower	0.735	0.791	0.855	0.851	0.900	0.895	0.833
	sign	0.691	0.756	0.809	0.826	0.856	0.967	1
	bird	0.629	0.689	0.786	0.801	0.829	0.898	0.666
	water	0.737	0.756	0.812	0.824	0.855	0.850	0.863
	book	0.708	0.752	0.823	0.826	0.860	0.855	0.857
	chair	0.658	0.715	0.764	0.805	0.786	0.782	0.796
	cat	0.769	0.835	0.900	0.886	0.922	0.900	0.800
	dog	0.764	0.831	0.896	0.864	0.935	0.872	0.444
	road	0.755	0.800	0.855	0.889	0.877	0.891	0.932
	boat	0.692	0.723	0.768	0.807	0.826	0.854	0.875

As shown in Table 4, compared with the other four algorithms, the CNN-TH method proposed in this study exhibits a noticeably higher annotation precision for most classes in the three datasets. For some classes with obvious features, such as desert and sunset in the MIML dataset, flower, bird and people in the COREL5K dataset, and tree and car in the MSRC dataset, the annotation precision is noticeably higher than other classes. For some classes, such as mountain, sea, beach, dinosaur, and cliff, the annotation precision is low due to the existence of similar features. For example, it is difficult to distinctly recognize two classes, cliff and mountain, and there exists a semantic gap between sea and beach owing to similar features. However, the annotation precision for the dog class in the MSRC dataset is the lowest, reaching only 44.4%, which may be attributed to the small number of image designs in the dog class and insufficient learning of the features for the dog class.

**Fig 5:**
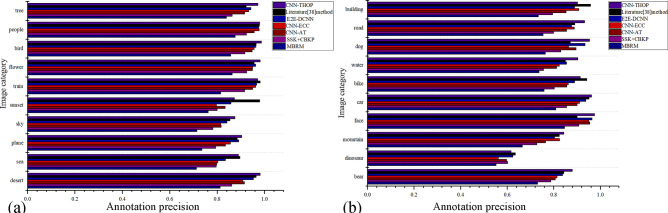
Experimental comparison of a single class precision using different algorithms.

In addition, we randomly select 2,000 pictures in 20 classes from the three datasets, i.e., MIML, COREL5K and MSRC, to constitute a new dataset for an experimental comparison of a single class accuracy, as shown in Fig 5.

As shown in Fig 5, the annotation precision based on the deep learning method is superior to that of the traditional algorithm, showing that the features extracted by the CNN are more comprehensive and approximate to people’s semantic understanding of images compared to those features extracted artificially. Compared with the E2E-DCNN and CNN-AT, the CNN-THOP proposed in this study improved the annotation precision for images of a single class. First, the CNN-THOP is based on the VGG16 model. By setting parameters and adjusting the network structure, the model structure is more applicable to the dataset in this study. Second, the model, by fusing the threshold optimization, sets an optimal threshold for each class to avoid omissions during the annotation process. Therefore, the annotation precision is significantly improved.

To verify the effectiveness of the CNN-THOP in automatic image labeling, we compare it with traditional methods, including the multiple Bernoulli relevance model (MBRM) and spatial spectrum kernel (SSK) combined with context-based keyword propagation (CBKP) (SSK+CBKP), as well as commonly used deep learning methods in recent years, such as the convolutional neural network and adaptive thresholding (CNN-AT) and the end-to-end automatic image annotation model based on a deep convolutional neural network (E2E-DCNN). The experimental results are shown in Table 5.

**Table 5:** Experimental results of various image annotation methods applied to different datasets.

Method	MIML				COREL5K				MSRC			
	*AP*	*AR*	*F1*	*CMD*	*AP*	*AR*	*F1*	*CMD*	*AP*	*AR*	*F1*	*CMD*
MBRM	0.532	0.556	0.543	0.356	0.240	0.250	0.245	0.201	0.428	0.452	0.440	0.356
SSK+CBKP	0.624	0.634	0.628	0.391	0.286	0.333	0.308	0.264	0.619	0.640	0.629	0.397
CNN-AT	0.732	0.749	0.740	0.436	0.317	0.373	0.343	0.297	0.718	0.832	0.771	0.428
CNN-ECC	0.736	0.758	0.747	0.452	0.379	0.437	0.406	0.318	0.706	0.747	0.726	0.435
E2E-DCNN	0.761	0.783	0.771	0.524	0.410	0.550	0.470	0.353	0.732	0.752	0.741	0.462
Literature [38]	0.780	0.796	0.788	0.576	0.497	0.559	0.526	0.381	0.729	0.763	0.746	0.538
CNN-THOP	0.807	0.776	0.787	0.648	0.527	0.583	0.553	0.412	0.761	0.783	0.771	0.586

As shown in Table 5, the CNN solutions are more suitable for multilabel annotation compared with the traditional machine learning methods, as the CNNs achieve noticeably better effects in terms of the investigated indexes. In the natural scene image MIML dataset, the average precision is improved by 27%, 18%, 7%, 7%, 4% and 2% compared to that of the other six methods. In the COREL5K dataset, the recall is improved by 33%, 25%, 21%, 15%, 3% and 2%. Compared with the MBRM model, the average precision and average recall of the CNN-THOP are improved by 34% and 33%, respectively, in the MSRC dataset, and its average recall is improved by 3%, compared with the E2E-DCNN. Overall, the proposed method in this study achieves the best performance on the MIML dataset. This is because fewer label classes are involved in this dataset compared with other datasets, which reduces the complexity. In contrast, the number of label categories in Corel5k reaches 260, which contains a large number of low-frequency labels. Therefore, the effect of the proposed method on this dataset is not satisfactory.

To further validate the effectiveness of the proposed CNN-THOP, pairwise *t*-tests are performed to assess the precision of the different methods over the three datasets. The results are shown in Table 6.

**Table 6:** Comparison of the precision of different methods over three datasets according to pairwise *t*-tests.

Comparison group	Mean ± standard deviation		Difference	*t*	*p*
MBRM vs. CNN-THOP	0.40±0.15	0.70±0.15	-0.30	-16.878	0.003**
SSK+CBKP vs. CNN-THOP	0.51±0.19	0.70±0.15	-0.19	-6.569	0.022*
CNN-AT vs. CNN-THOP	0.59±0.24	0.70±0.15	-0.11	-2.137	0.166
CNN-ECC vs. CNN-THOP	0.61±0.20	0.70±0.15	-0.09	-3.182	0.086
E2E-DCNN vs. CNN-THOP	0.63±0.19	0.70±0.15	-0.06	-2.375	0.141
Literature [38] vs. CNN-THOP	0.67±0.15	0.70±0.15	-0.03	-20.418	0.002**

*p<0.05

**p<0.01

As shown in Table 6, the CNN-THOP significantly improves the annotation precision for the three datasets compared with the MBRM, SSK+CBKP and the method used in the literature [38] (*p*<0.05). Although the annotation precision of the CNN-THOP does not show a significant difference compared with the CNN-AT, CNN-ECC and E2E-DCNN, the precision increases by 11%, 9% and 6%, respectively. These findings indicate that the proposed method in this study is effective for multilabel image annotation.

**Fig 6:**
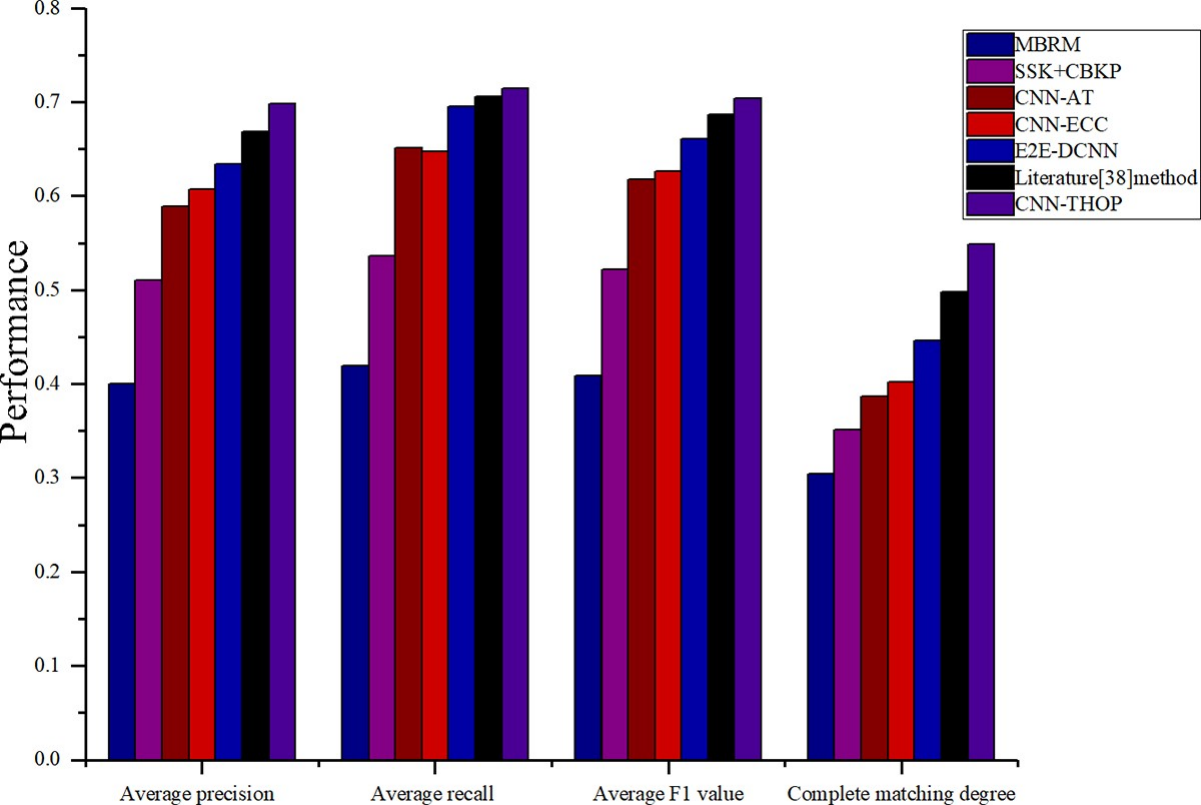
Comparison of evaluation indexes between different algorithms and different datasets.

In addition, we mix MIML, COREL5K and MSRC to form a large dataset containing 7,591 images in 287 classes and conduct an experimental comparison in terms of average precision, average recall, *F1* value and *CMD*, as shown in Fig 6.

As shown in Fig 6, the annotation precisions for the CNN-AT, the E2E-DCNN, and the CNN-THOP proposed in this study are much higher than those of the traditional methods, i.e., the MBRM and SSK+CBKP, indicating that at present, the deep learning method in the field of image labeling is superior to the traditional machine learning method. This once again demonstrates that CNNs are superior to artificial methods for feature extraction. Therefore, the annotation precision is significantly higher than that in traditional methods. As shown in Fig 6, of the four evaluation indexes, *CMD* is the lowest because *CMD* is a more rigorous evaluation index. *CMD* indicates that neither excessive labeling nor labeling omissions occur during the labeling process, thereby realizing accurate labeling and verifying the effectiveness of the method in this study once again.

### Actual labeling effect of the model

**Fig 7:**
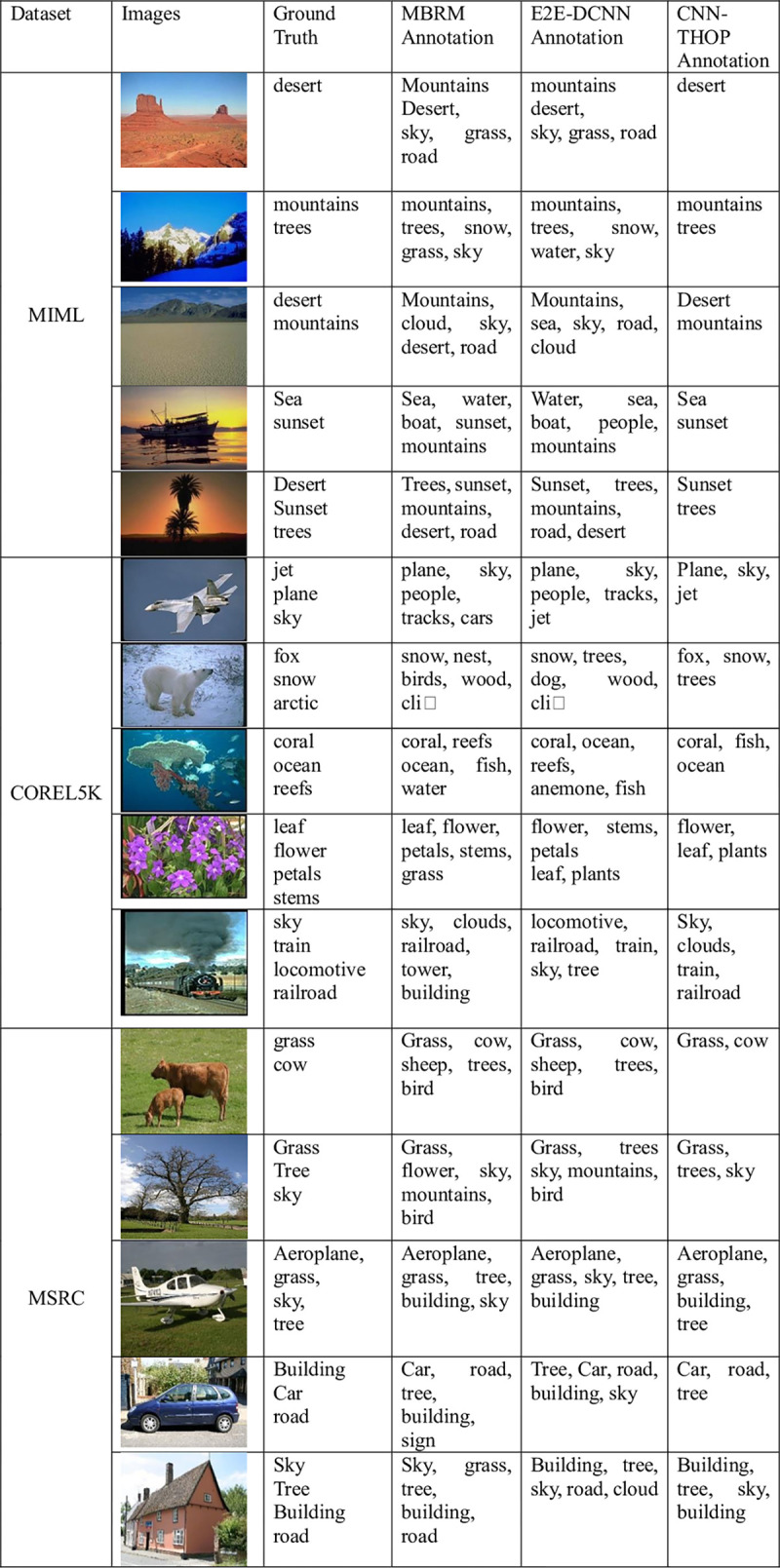
Comparison of image annotation results using various experimental methods.

Fig 7 shows the actual effect of automatic labeling for various experimental methods, in which the traditional MBRM and the deep learning E2E-DCNN are selected for comparison with the prediction labels in this study.

As shown in Fig 7, the method proposed in this study is more effective than other methods in automatic image annotation, as most images can be annotated with completely correct labels without the issue of multiple or fewer labels. Compared with other methods, this method solves the problem caused by a fixed number of labels. The problem of individual wrong labels or fewer labels may be caused by the fact that the number of images for the class in question is too small and the model does not fully learn this feature. For the labeling results of the traditional MBRM and the deep learning E2E-DCNN, it is clear that there are numerous problems with overlabeling or downlabeling caused by the fixed number of labels, making it difficult to achieve precise annotation. Overall, the method in this study is more accurate and effective at automatic image annotation.

## Conclusions

To address a fixed number of labels appearing during the multilabel image annotation process and label annotation according to the ranking function, we propose in this study the application of a CNN-THOP for image annotation. First, a CNN model is used to predict the probability for each class of labels. Due to the merits of the VGG16 network architecture, we improved the CNN structure in this study based on VGG16. A BN added within the CNN significantly accelerates the convergence speed, and the network structure and parameters are adjusted to make them more suitable for the datasets in this study. Next, a threshold optimization algorithm finds an optimal threshold for each class of labels. Finally, only when the prediction probability of labels of a class in question is greater than or equal to the optimal threshold will this class of labels be assigned. The setting of the optimal threshold solves the problem of overlabeling or downlabeling caused by a fixed number of labels, making labeling more rational and effective. The experimental results for three public datasets—MIML, COREL5K and MSRC—indicate that the average precision of the CNN-THOP is 80.7%, 52.7% and 76.1%, respectively, and the average recall is 77.6%, 58.3% and 78.3%, respectively. The *F1* value reaches 78.7%, 55.3% and 77.1%, respectively, and the *CMD* also reaches 64.8%, 41.2% and 58.6%, respectively. Compared with other methods, the parameters in this study are greatly improved, demonstrating that the method proposed in this study can be used for effective image annotation. The deficiency of this study is that the improved VGG16 model is a shallow CNN and cannot fully extract higher-level image features, and its accuracy declines in terms of the prediction probability, resulting in deviations in subsequent threshold optimization. A future study will be carried out to examine two factors. 1) In terms of feature extraction, the artificial feature extraction and convolution operation will be combined to perfect the feature extraction and avoid omissions. We will further deepen the CNN structure in this study and extract higher-level features with reference to the characteristics of VGG16. 2) We will optimize the threshold optimization algorithm to make it more reasonable and efficient to find the optimal threshold for each class of labels.
